# Utility of routine chest radiographs after chest drain removal in paediatric cardiac surgical patients—a retrospective analysis of 1076 patients

**DOI:** 10.1093/icvts/ivad159

**Published:** 2023-09-26

**Authors:** Gautham Shetty, Jason Zouki, Geraldine Lee, Aditya Patukale, Kim S Betts, Robert N Justo, Supreet P Marathe, Prem Venugopal, QPCR Group, Jessica Suna, Tom Karl, Nelson Alphonso

**Affiliations:** Queensland Paediatric Cardiac Service (QPCS), Queensland Children’s Hospital, Brisbane, QLD, Australia; School of Medicine, Children's Health Queensland Clinical Unit, University of Queensland, Brisbane, QLD, Australia; Queensland Paediatric Cardiac Research, Children’s Health Queensland, Brisbane, QLD, Australia; School of Medicine, University of Queensland, Brisbane, QLD, Australia; School of Medicine, University of Queensland, Brisbane, QLD, Australia; Queensland Paediatric Cardiac Service (QPCS), Queensland Children’s Hospital, Brisbane, QLD, Australia; School of Medicine, Children's Health Queensland Clinical Unit, University of Queensland, Brisbane, QLD, Australia; Queensland Paediatric Cardiac Research, Children’s Health Queensland, Brisbane, QLD, Australia; School of Public Health, Curtin University, Perth, WA, Australia; Queensland Paediatric Cardiac Service (QPCS), Queensland Children’s Hospital, Brisbane, QLD, Australia; School of Medicine, Children's Health Queensland Clinical Unit, University of Queensland, Brisbane, QLD, Australia; Queensland Paediatric Cardiac Research, Children’s Health Queensland, Brisbane, QLD, Australia; Queensland Paediatric Cardiac Service (QPCS), Queensland Children’s Hospital, Brisbane, QLD, Australia; School of Medicine, Children's Health Queensland Clinical Unit, University of Queensland, Brisbane, QLD, Australia; Queensland Paediatric Cardiac Research, Children’s Health Queensland, Brisbane, QLD, Australia; Queensland Paediatric Cardiac Service (QPCS), Queensland Children’s Hospital, Brisbane, QLD, Australia; School of Medicine, Children's Health Queensland Clinical Unit, University of Queensland, Brisbane, QLD, Australia; Queensland Paediatric Cardiac Research, Children’s Health Queensland, Brisbane, QLD, Australia; Queensland Paediatric Cardiac Service (QPCS), Queensland Children’s Hospital, Brisbane, QLD, Australia; School of Medicine, Children's Health Queensland Clinical Unit, University of Queensland, Brisbane, QLD, Australia; Queensland Paediatric Cardiac Research, Children’s Health Queensland, Brisbane, QLD, Australia

**Keywords:** Chest drain, Chest radiographs, Paediatric cardiac surgery

## Abstract

**OBJECTIVES:**

Chest drains are routinely placed in children following cardiac surgery. The purpose of this study was to determine the incidence of a clinically relevant pneumothorax and/or pleural effusion after drain removal and to ascertain if a chest radiograph can be safely avoided following chest drain removal.

**METHODS:**

This single-centre retrospective cohort study included all patients under 18 years of age who underwent cardiac surgery between January 2015 and December 2019 with the insertion of mediastinal and/or pleural drains. Exclusion criteria were chest drain/s in situ ≥14 days and mortality prior to removal of chest drain/s. A drain removal episode was defined as the removal of ≥1 drains during the same episode of analgesia ± sedation. All chest drains were removed using a standard protocol. Chest radiographs following chest drain removal were reviewed by 2 investigators.

**RESULTS:**

In all, 1076 patients were identified (median age: 292 days, median weight: 7.8 kg). There were 1587 drain removal episodes involving 2365 drains [mediastinal (*n* = 1347), right pleural (*n* = 598), left pleural (*n* = 420)]. Chest radiographs were performed after 1301 drain removal episodes [mediastinal (*n* = 1062); right pleural (*n* = 597); left pleural (*n* = 420)]. Chest radiographs were abnormal after 152 (12%) drain removal episodes [pneumothorax (*n* = 43), pleural effusion (*n* = 98), hydropneumothorax (*n* = 11)]. Symptoms/signs were present in 30 (2.3%) patients. Eleven (<1%) required medical management. One required reintubation and 2 required chest drain reinsertion.

**CONCLUSIONS:**

The incidence of clinically significant pneumothorax/pleural effusion following chest drain removal after paediatric cardiac surgery is low (<1%). Most patients did not require reinsertion of a chest drain. It is reasonable not to perform routine chest radiographs following chest drain removal in most paediatric cardiac surgical patients.

**Figure:**
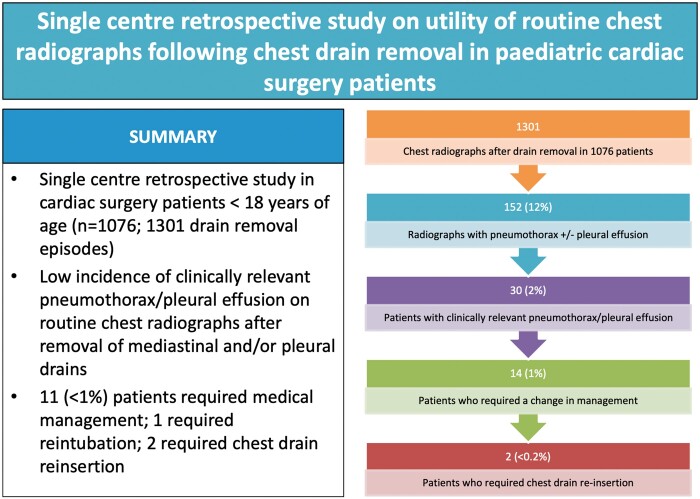

## INTRODUCTION

Chest drains are routinely placed in children following cardiothoracic surgery to decrease potential post-operative complications, principally pneumothoraces and pleural effusions. Chest radiographs are the standard diagnostic modality for pneumothorax with a reported sensitivity of 52% and specificity of 100% [1]. In most institutions it is considered routine practice to perform chest radiographs after chest drain removal [2]. However, previous studies have questioned the utility of these protocols, considering the potential impact on clinical management by evaluating major re-interventions, radiation exposure to the patient and total cost-effectiveness following chest drain insertion intraoperatively [1–11]. Cumulative low dose ionizing radiation has been independently associated with cancer and can predispose the most complex patients including those with congenital heart disease to an increased risk of developing cancer as adults [12, 13]. As the number of safe exposures is not known and the lifetime risk is cumulative, children should ideally avoid having any unnecessary radiographs.

A best evidence report predominantly in adult cardiothoracic patients reported the detection of pneumothoraces and effusions in the range of 2–40% on routine chest radiographs following chest drain removal (level 3 evidence) [2]. Studies suggest that significant pneumothoraces can be recognized from symptoms and clinical signs including abnormal arterial blood gases, haemodynamic instability, dyspnoea, low haemoglobin oxygen saturations and abnormal findings on physical examination. Studies report that whilst clinical evaluation is less sensitive than chest radiography, clinicians have an increased specificity of up to 79% for detecting mild-moderate complications after chest drain removal. Importantly, even in patients with confirmed complications after chest drain removal, only 1.5% require clinical intervention [11].

There have been limited studies in children which have shown that close clinical observation is sufficient to detect a clinically relevant pneumothorax after chest drain removal [6, 7, 10]. However, the data with respect to the paediatric cardiac surgical population are insufficient to conclusively recommend the elimination of a routine chest radiograph after chest drain removal. We therefore decided to conduct a retrospective study to determine whether a chest radiograph can be safely avoided following chest drain removal.

We aimed to assess the utility of chest radiographs after chest drain removal by determining the incidence of pneumothorax/pleural effusion following chest drain removal and whether a radiographically defined pneumothorax (or pleural effusion) could have been identified clinically in these patients.

## PATIENTS AND METHODS

### Ethics approval

The study was approved by the hospital Human Research Ethics Committee (HREC reference number: LNR/20/QCHQ/64265).

The Human Research Ethics Committee granted a waiver of consent as the project met the waiver requirements defined in the National Statement on Ethical Conduct in Human Research, 2007 (updated 2018), the National Health and Medical Research Council, the Australian Research Council and Universities Australia, Commonwealth of Australia, Canberra.

### Study design

A retrospective, cohort study was conducted at a quaternary, paediatric hospital providing a state-wide paediatric cardiac surgical service. Patients were identified from routinely populated departmental and hospital databases. Demographic and clinical data were extracted from the hospital electronic medical record. Chest radiographs following chest drain removal were independently reviewed by 2 investigators.

### Inclusion criteria

All patients between 0 days and 18 years of age who underwent cardiac surgery with insertion of mediastinal and/or pleural drains over a 5-year period between 1 January 2015 and 31 December 2019.

### Exclusion criteria

1. Chest drain/s in situ ≥14 days (as these patients often develop an enlarged drain insertion site with or without suture dislodgement and are at higher risk of pneumothorax post-drain removal).
2. Mortality prior to removal of chest drain/s.

### Definitions and technique of drain removal

Drain removal episode was defined as removal of ≥1 drains during the same episode of analgesia ± sedation. All chest drains were removed by 2 registered nurses on-duty for that shift and/or cardiac surgical fellows using a standard protocol (Supplementary Material).

Following drain removal, patients were closely monitored for 4 h and were specifically assessed for signs of respiratory distress including increased work of breathing, tachypnoea, dyspnoea, decrease in haemoglobin oxygen saturations, decreased chest movement and/or breath sounds and increasing pain.

### Statistical analysis

Continuous variables were summarized as median [interquartile range (IQR)] and categorical variables as proportions (frequency). Due to the non-normal distribution of the continuous variables included in the analysis, the Kruskal–Wallis test was used to compare the distributions of these across the patient groupings of chest radiograph outcomes. The small number of observations in some groups (i.e. 3 in 1 group and 11 in another) precluded the use of the asymptotic *P*-value, whilst the presence of many ties in the data also meant the exact test could not be calculated. Thus, we used a permutations approach, which relied on Monte Carlo resampling to approximate the enumeration of all permutation samples in the data. Fisher exact tests were used to compare categorical variables by groups. All analyses were undertaken in R (open-source programming language for statistical computing available under the GNU General Public License; https://www.r-project.org).

### Cost analysis

The local cost of a chest radiograph, total chest radiographs performed and the incidence of drain removal episodes with clinical examination supporting the diagnosis of a pneumothorax/pleural effusion were used to calculate potential cost savings by avoiding routine radiographs in asymptomatic patients.

## RESULTS

### Patient population

Over the study period, 1178 patients were identified who underwent 1397 cardiac procedures. Of these, 102 patients were excluded from the study [no chest drains inserted (*n* = 64), drain removals >14 days after the procedure (*n* = 25), mortality prior to removal of chest drains (*n* = 13)]. Thus, 1076 patients who underwent 1270 procedures were included in the study. Baseline characteristics of all patients are presented in Tables 1–3. The median age was 292 days (IQR 62–1956) and the median weight was 7.8 kg (IQR 4.1–18.5). Chest drains remained in situ for a median of 3.0 days (IQR 2.0–5.0). Patients who developed a pneumothorax and/or pleural effusion post-chest drain removal were older with a higher median weight. There was no difference in age and weight between patients with a normal chest radiograph post-chest drain removal and those in whom specific additional management or intervention was required after chest drain removal (Table 3). Patients who required intervention after chest drain removal had a longer intensive care unit stay (Table 3).

**Table 1:** Demographic and clinical characteristics

Variable	*n* (%) or median (IQR)
Total patients	1076
Total procedures	1270
Age (days)	292 (62–1956)
Weight (kg)	7.8 (4.1–18.5)
Males	700 (55%)
Additional non cardiac diagnosis	216 (17%)
Sternotomy	1190 (94%)
Use of cardiopulmonary bypass	1176 (93%)
CPB time (min)	90.5 (54–152)
Cross-clamp time (min)	62 (34–106)
Aristotle score	10 (7.5–12)
Duration of ventilation (hours)	17 (9–48)
ICU stay (days)	2 (1–5)
Hospital stay (days)	11 (6–24)
Duration of chest drains (days)	3 (2–5)

CPB: cardiopulmonary bypass; ICU: intensive care unit; IQR: interquartile range.

**Table 2:** Diagnosis subgroups—all procedures (*n* = 1270)

Diagnosis	% (*n*)
Septal defects	25% (321)
Right heart lesions	19% (239)
Thoracic arteries and veins	15% (192)
Left heart lesions	14% (181)
Transposition of the great arteries	7% (89)
Single ventricle	6% (79)
Pulmonary venous anomalies	5% (57)
Double outlet right ventricle	4% (48)
Miscellaneous	5% (64)

**Table 3:** Comparison of baseline data between groups

	Normal chest radiograph (1112 patients)	Abnormal chest radiograph (158 patients, 12%)	Non-invasive measures (11 patients, 8%)	Intervention (3 patients)	*P*-Value
Median age (days)	238 (42–1701)	1602 (154–3558)	337 (115–3556)	229 (120–798)	**<0.001**
Median weight (kg)	7.0 (3.9–16.6)	17.2 (5.8–32.2)	9.0 (5.0–32.0)	6.7 (4.9–10.9)	**<0.001**
Gender, male, % (*n*)	56% (628)	51% (81)	72% (8)	0% (0)	0.088
Non cardiac diagnosis, % (*n*)	17% (168)	21% (30)	19% (3)	33% (1)	0.195
Sternotomy, % (*n*)	93% (1031)	95% (150)	100% (11)	100% (3)	0.633
Cardiopulmonary bypass, % (*n*)	89% (990)	92% (146)	100% (11)	100% (3)	0.496
CPB time (min), median (IQR)	102.0 (52.0–162.0)	97.0 (65.0–150.0)	106.0 (49.0–126.0)	105.0 (96.0–108.5)	0.959
Cross-clamp time (min), median (IQR)	62.0 (27.0–107.0)	58.0 (26.0–98.5)	67.0 (0.0–77.0)	66.0 (62.0–80.5)	0.368
Aristotle score	10.5 (8.0–13.0)	11.0 (9.0–12.3)	11.0 (9.8–12.0)	9.8 (7.4–10.2)	0.367
Duration of ventilation (h), median (IQR)	24.0 (10.0–60.0)	12.0 (8.5–30.0)	15.5 (10.25–34.3)	16.0 (57.5–174.5)	**0.009**
Length of ICU stay (days), median (IQR)	3.0 (1.0–7.0)	2.0 (1.0–6.0)	2.0 (1.0–4.5)	12.0 (11.0–13.0)	**0.009**
Length of hospital stay (days), median (IQR)	14.0 (7.0–28.0)	10.0 (6.0–23.0)	15.0 (9.0–41.0)	17.0 (12.0–58.0)	0.064
Duration of chest drains (days), median (IQR)	3.0 (2.0–5.0)	3.0 (2.0–5.0)	3.0 (2.0–3.0)	6.0 (4.0–8.0)	0.514

Differences between groups on continuous variables were examined using the Kruskal–Wallis test with Monte Carlo resampling. Fisher exact tests were used to compare categorical variables by groups.

CPB: cardiopulmonary bypass; CXR: chest radiograph; ICU: intensive care unit; IQR: interquartile range.

p value in bold indicates statistical significance.

### Drain removal episodes

There were 1587 drain removal episodes (1 or more drains were removed during each episode) which involved the removal of 2365 drains. Of these, 1347 (57%) were mediastinal drains, 598 (25%) were right pleural drains and 420 (18%) were left pleural drains. There was no mortality associated with a drain removal episode.

### Chest radiographs

Chest radiographs were performed following 1301 (*n* = 1587; 82%) drain removal episodes. No chest radiographs were performed following removal of 286 (18%) drains, of which 285 drains were mediastinal and 1 was a right pleural drain. These drain removal episodes were excluded from further analysis.

**Figure 1:**
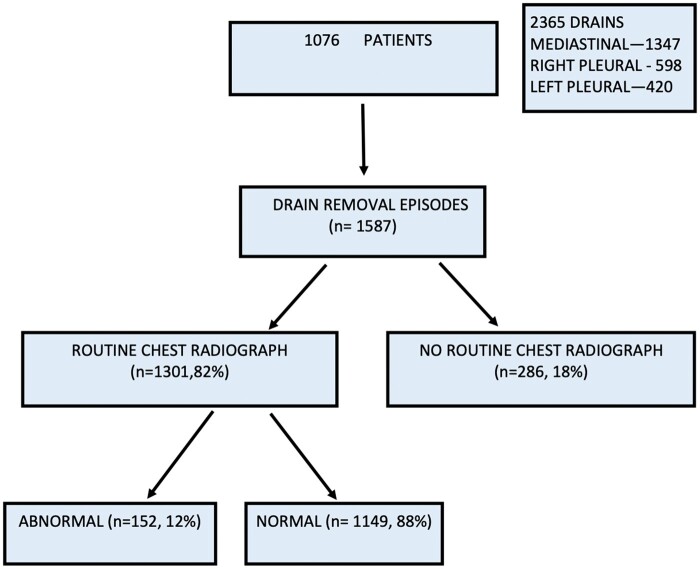
Study participants.

**Figure 2:**
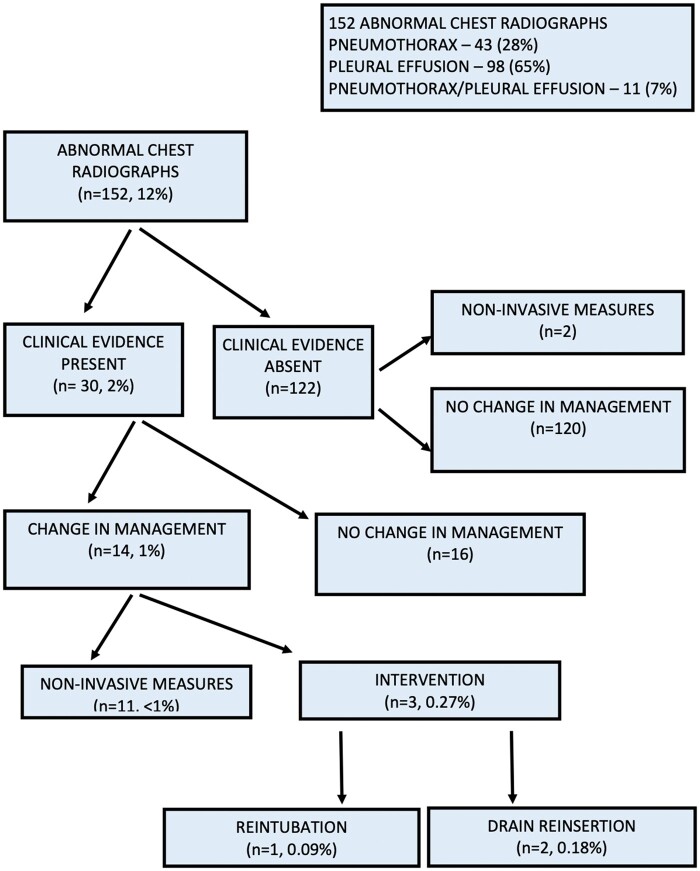
Abnormal chest radiographs following chest drain removal (*n* = 152, 12%).

The chest radiograph did not demonstrate the presence of a pneumothorax or pleural effusion after 1149 (*n* = 1301; 88%) drain removal episodes (Fig. 1). The chest radiograph was abnormal after 152 (12%) drain removal episodes [pneumothorax after 43 episodes (28%), pleural effusion after 98 episodes (65%) and pneumothorax and pleural effusion after 11 episodes (7%)] (Fig. 2). Clinical signs or symptoms were present after 30 (2%) drain removal episodes with an abnormal chest radiograph. Of these, only 14 (1%) patients required specific additional management. Eleven (<1%) patients required only medical management i.e. increased oxygen delivery via nasal prongs/mask ventilation or diuresis. One patient required reintubation and ventilation and chest drain reinsertion. Two patients required only chest drain reinsertion. Overall, the incidence of early chest drain reinsertion after chest drain removal in the 1076 patients was 0.18% (*n* = 2).

### Cost analysis

A within‐study cost analysis was completed using the Queensland Health cost base. The number of mobile chest radiographs that could have been avoided using clinical evidence of pneumothorax/pleural effusion as an indicator for requirement for intervention was multiplied by the average cost per mobile chest radiograph ($47.15). In our study, a total of 1301 routine chest radiographs were performed after chest drain removal. Fourteen patients developed a pneumothorax/pleural effusion that required medical or surgical intervention, all of whom had clinical evidence of the pneumothorax/pleural effusion. Thus potentially 1271 chest radiographs could have been avoided during the study period resulting in potential cost savings of A$59 928 (A$47.15 × 1271).

## DISCUSSION

Our study comprising >1000 paediatric cardiac surgical patients showed that the incidence of clinically significant pneumothorax/pleural effusion following chest drain removal was low (<1%). There was no mortality related to chest drain removal. Most patients who developed a post-chest drain removal pneumothorax were managed medically and did not require chest drain reinsertion. Only 3 patients required reinsertion of the chest drain due to a large pneumothorax.

Chest radiographs are often routinely performed after removal of chest drains to assess for a possible pneumothorax [14]. However, some studies in postoperative cardiac patients have challenged the use of routine radiographs following chest drain removal, suggesting that only clinically indicated chest radiographs should be obtained. Most of these studies have been in adult cardiac and non-cardiac patients [1–5, 8, 9]. There is limited evidence specific to the paediatric cardiac surgery population [7, 15].

Eighteen percentage of patients in our study did not have a routine chest radiograph following drain removal. Seventeen percentage of the patients had only a mediastinal drain removed which are usually not associated with post-drain removal pneumothorax, unless either pleura is breached. No adverse sequelae were documented for these patients.

The majority (88%) of the patients in our study had normal postoperative findings on the routine chest radiographs performed after chest drain removal. Twelve percentage of the patients in our study who had routine chest radiograph post-drain removal had abnormal findings of pneumothorax, pleural effusion, or both. Sepehripour *et al.* presented 6 best evidence studies (level 3 evidence) which reported the detection of pathology on routine chest radiographs ranging from 2% to 40% compared with 79% in clinically indicated chest radiographs. Whilst the incidence of intervention following routine chest radiographs was as high as 4% in the smallest study, clinical symptoms and signs were a significant predictor of major reintervention. The authors concluded that routine post-drain removal chest radiography provides no diagnostic or therapeutic advantage over clinically indicated chest radiography or simple clinical assessment and that the omission of routine post-drain removal chest radiographs in patients following cardiothoracic surgery is safe [2]. Our study supports these conclusions.

In our study, 12% of patients had an abnormal chest radiograph following chest drain removal. Most of these patients (92%) were asymptomatic and did not require any additional intervention. The remaining 8% of patients who required additional medical or surgical intervention had clinical symptoms and signs consistent with a large pneumothorax.

The incidence of immediate chest drain reinsertion after chest drain removal in our study was <1% which was slightly lower than that reported by McCormick *et al.* [5] (1.4% incidence of intervention post-removal of chest drains).

Previous studies have described signs and symptoms which should be used for close monitoring after chest drain removal. These include increased work of breathing, changes in heart rate and blood pressure, patient discomfort, chest pain, tachycardia, dyspnoea, altered respiratory rate, increased oxygen requirement to maintain haemoglobin oxygen saturations, worsening acidosis, decreased breath sounds, tracheal deviation, hyperresonance, haemodynamic instability, new onset restlessness, anxiety and agitation [11]. Cunningham *et al.* [3] identified dyspnoea as the most common clinical symptom precipitating chest drain reinsertion, followed by low haemoglobin oxygen saturations and decreased breath sounds. Pacharn *et al.* noted that clinical signs were noted in 6 of 7 (85.7%) patients out of 374 paediatric cardiac patients, who later underwent a major clinical intervention. Only 1 patient who had a drain reinserted for a moderate pneumothorax was asymptomatic [7]. This is similar to the findings in our study.

van den Boom and Battin have previously demonstrated a pneumothorax after 9 of 35 (26%) drain removal episodes which is higher than the proportion of patients in our study. However, none of the patients in their study required reintervention [10]. In a study by Eisenberg and Khabbaz [4], 4 patients (4.2%) developed a pneumothorax requiring post-chest drain management (1 showed clinical signs requiring drain re-insertion and 1 required re-insertion for pleural effusion) after 95 chest drain episodes. This is similar to the findings in our study.

At a local cost of A$47.15 per chest radiograph in our hospital, avoiding routine radiographs in asymptomatic patients would have resulted in a potential cost saving of A$59 928. This is comparable to the calculations by McCormick *et al.* [5] which stated that omission of postoperative chest drain removal chest radiographs for 283 patients saved US$31 696.

### Limitations

Our study has all the limitations of a retrospective study and as there was no control group, the study design did not permit a comparison of outcomes of clinical examination alone with those after routine chest radiographs. This study assumes that chest radiographs, whilst being the gold-standard for diagnosis and monitoring of a pneumothorax, have a sensitivity of 100%. Chest drain removals were performed by different nurses/surgical fellows, and subtle differences in technique and expertise may have influenced outcomes. There is a varied approach amongst clinicians with respect to the timing of chest tube removal which could also influence outcomes. We have reported the results from a single institution and the protocols for the removal of drains as well as the performance of chest radiographs may be different in other institutions. Consequently, the results are not generalizable. We did not collect information about whether patients were intubated and ventilated at the time of drain removal. However, drain re-insertion was not required in any patient who was intubated and ventilated at the time of drain removal in this study population. Though we recommend reliance on clinical signs and symptoms to determine the need for a chest radiograph, the patient physiology and operation performed as well as the skill and experience of the clinical staff monitoring the patient needs to be taken into consideration. Our cohort includes patients of varying age, diagnosis and complexity. The implications of a missed pneumothorax or collapsed lung will be far more severe in patients with reduced physiological reserve such as those with single ventricle physiology, prematurity or pulmonary comorbidities. In such patients, it may be safer to do a chest radiograph after removal of a chest drain.

## CONCLUSION

The incidence of clinically significant pneumothorax/pleural effusion following chest drain removal after paediatric cardiac surgery is low (<1%). There was no mortality related to chest drain removal. Most patients with chest radiograph findings were managed medically and did not require chest drain reinsertion. It is reasonable not to perform routine chest radiographs after chest drain removal in most paediatric cardiac surgical patients. However, firm recommendations can only be made after a prospective randomized study comparing the outcomes of clinical examination with chest radiographs after drain removal.

## Supplementary Material

ivad159_Supplementary_Data

## Data Availability

The data underlying this article will be shared on reasonable request to the corresponding author.

## Notes

Presented on 10 May 2022 at the Society of Cardiothoracic Surgeons in Great Britain and Ireland (SCTS) Annual Meeting, Belfast, Ireland, 2022.

## ABBREVIATION

IQR: Interquartile range
