# Supplementary material for: Utility of routine chest radiographs after chest drain removal in paediatric cardiac surgical patients—a retrospective analysis of 1076 patients
Source: Interdiscip Cardiovasc Thorac Surg. 2023 Sep 26;37(4):ivad159. doi: 10.1093/icvts/ivad159 (PMC10560101; doi:10.1093/icvts/ivad159)
Supplement: ivad159_Supplementary_Data [file ivad159_supplementary_data.docx]

**Chest drain removal protocol – Queensland Children’s Hospital Guidelines**

*Equipment*

Dressing pack, sterile gloves x 2 pairs, personal protective equipment, stitch cutter, normal saline, steri-strips, and analgesia +/- sedation

*Coagulation*

It is not usual to stop prophylactic heparin (10 units/kg/h) prior to chest drain removal. However, due consideration is given for a brief cessation of heparin (balance risk vs benefit) when a patient is therapeutically anticoagulated and/or when the drain has been in situ for a prolonged period. Coagulation studies are considered in patients who are anticoagulated or coagulopathic, prior to drain removal.

*Procedure*

Drain removal is a two-person procedure. The procedure is explained to patient and parents. Analgesia +/- sedation is provided in advance if required (e.g., narcotic bolus as ordered). Equipment is prepared followed by handwashing. The Drains can be left on suction for removal. Multiple drains that are connected will be clamped to be removed. Where possible the drains are separated. Suction is reinstated on any drains that are to remain in situ. Only the drain to be removed is clamped. After donning sterile gloves, the twirled purse string suture if present is located and untwirled until the two ends are free. A knot is loosely tied when ready for drain removal. The drain site is swabbed with normal saline to cleanse the site and remove old blood. The anchoring suture is cut and removed (ensuring purse string suture is not cut). In a spontaneously breathing patient – the child is instructed to take a deep breath and hold while drain is removed. In a ventilated patient the drain is removed on peak inspiration. The drain is pulled out with the assistant holding (pinching) the wound edges together. If present, the purse string suture is pulled to gather in skin edges and close the wound. The purse string is tied with two to three knots. Excess purse string material is cut off leaving approximately two centimetres to enable suture removal later. In the absence of a purse string, the drain site is covered with a gauze square immediately to remove ooze and any remaining clot. Whilst maintaining pinch; the wound edges are secured with steri-strips, using one steri-strip perpendicularly across the wound and then several in a crisscross pattern. For drain sites that ooze after drain removal, the use of steri-strips to seal skin edges may be ineffective. In that situation steri-strips, dry gauze and occlusive dressings are applied over the site until fluid loss has ceased. The used equipment is discarded appropriately. Personal Protective Equipment is removed, and hands are washed. A chest x-ray may be required post procedure in consultation with the cardiac surgical and paediatric intensive care unit teams and reviewed by a medical officer. The events are then documented.
